# Depressive symptoms and self-rated health among Brazilian older adults: baseline data from the ELSI-Brazil study

**DOI:** 10.47626/1516-4446-2023-3331

**Published:** 2024-03-01

**Authors:** Natalia T. Ito, Déborah Oliveira, Fabricio M.S. Rodrigues, Erico Castro-Costa, Maria F. Lima-Costa, Cleusa P. Ferri

**Affiliations:** 1 Departamento de Psiquiatria, Universidade Federal de São Paulo, São Paulo, SP, Brazil; 2 Facultad de Enfermería, Universidad Andrés Bello, Campus Viña del Mar, Chile; 3 Millenium Institute for Care Research (MICARE), Santiago, Chile; 4 Núcleo de Estudos em Saúde Pública e Envelhecimento, Fundação Oswaldo Cruz, Belo Horizonte, MG, Brazil; 5 Universidade Federal de Minas Gerais, Belo Horizonte, MG, Brazil

**Keywords:** Self-rated health, depressive symptom, depression, older adult, health

## Abstract

**Objective:**

To investigate whether having a higher number of depressive symptoms is associated with negative self-rated health (SRH) even in the absence of illness.

**Methods:**

This is a secondary analysis of baseline data from the Brazilian Longitudinal Study of Aging (ELSI-Brazil), conducted in 2015-2016, using a national sample of 9,412 people aged 50 or over. SRH was dichotomized into poor or very poor and very good or excellent, good, or average. Depressive symptoms were assessed through the eight-item Center for Epidemiologic Studies Depression Scale (CES-D8). Sociodemographic variables, information about unhealthy behaviors, and the number of chronic conditions were also analyzed.

**Results:**

Having depressive symptoms was strongly associated with poor or very poor SRH both in the unadjusted and adjusted analyses. The magnitude of the association was reduced when the number of chronic illnesses was included in the multivariate analysis, along with the other sociodemographic variables and unhealthy behaviors (OR 1.35, 95%CI 1.31-1.39).

**Conclusion:**

Having depressive symptoms may contribute towards having a poorer perception of health, even in the absence of health conditions. SRH is a multidimensional construct that can accurately reflect a person’s state of general mental health.

## Introduction

Self-rated health (SRH) is an indicator of general health which is widely used in gerontological research, and refers to a person’s own health perception.^1^ Poor SRH has been found to be an independent predictor of mortality and functional decline among clinical populations^2^ and is strongly associated with a higher frequency of medical appointments and utilization of health care services when compared to objective measures of health status.^3^ In addition, it is an idiosyncratic and subjective perception connected to a given social and cultural context.^4^ It can be determined by asking a simple question – “In general, how would you describe your general health?” – and reflects the individual’s integrated perception of themselves, including both objective aspects of health (current physical state), health behaviors, subjective assessment of symptoms, and psychological aspects, that is, interpretations and perceptions about one’s own health.^5^ However, in clinical contexts, positive perceptions of SRH in a person with medical conditions is often interpreted by clinicians as being a denial of illness, while a negative assessment of SRH in an objectively healthy person may be linked to depressive symptoms.^6^

Depression is a common health problem among older people and is associated with a significant loss of physical function and quality of life, affecting patients, their families, and society.^7^ According to the 2017 Global Burden of Disease (GBD) study, depression is ranked fourth among the 10 most common health problems in Brazil.^8^ Psychological mechanisms of depression, which include expectations and interpretations, may play an important role in explaining the potential effect of depressive symptoms on SRH.^9^ Late-life depression usually occurs along with several chronic medical conditions, is associated with inflammatory activity, and is often attributed to the aging process itself or seen as a normal response to losses or illnesses.^10^ The comorbid state of depression and chronic organic illness worsens health in an additive and synergistic way compared to having depression alone without chronic illnesses or chronic illnesses alone without depression. From a biological point of view, objective threats to health, such as heart disease, cancer, or physical limitations, can increase one’s depressive state.^11^ Given that SRH includes assessments of both physical and psychological elements related to health threats, it is expected to affect depressive symptoms. Most studies on this topic have used a cross-sectional design and have found an association between SRH and depressive symptoms but could not establish whether depressive symptoms lead to poor SRH or poor SRH leads to depressive symptoms.^1^,^9^,^12^,^13^ The importance of carrying out a nationwide study using a representative sample of the Brazilian older population is highlighted by the increasing prevalence of depression in this population and its consequences on overall health status. The Brazilian Longitudinal Study of Aging (ELSI-Brazil) is the largest population-based study conducted in Brazil to date. It covers all five geographic regions of the country and, therefore, allows for a more robust assessment of the association between SRH, depression, and other variables compared to previous, smaller studies. The present investigation was designed to ascertain whether depressive symptoms are significantly related to poorer perceived health status independently of actual physical morbidity and to analyze other factors related to SRH among Brazilian older adults.

## Methods

### Study design and sample

This is a secondary analysis of the baseline data of ELSI-Brazil, collected in 2015-2016. ELSI-Brazil is a household-based survey conducted in a nationally representative sample of residents of 70 municipalities distributed across the five geographic regions of Brazil. All national household surveys conducted in Brazil use the Instituto Brasileiro de Geografia e Estatística (IBGE) geographic operational database for stratification and selection of sampling areas. To ensure that the sample represents the urban and rural areas of the small, medium, and large municipalities included, the ELSI-Brazil sampling strategy used a design with selection stages, combining stratification of primary sampling units (municipalities), census tracts, and households. All individuals aged 50 years and older living in the selected households, were eligible for interviews and other study procedures. The final sample included 9,412 participants. Other methodological details have been published elsewhere.^14^

### Measures

#### Depressive symptoms

Depressive symptoms were assessed using the eight-item Center for Epidemiologic Studies Depression Scale (CES-D8). This is an abbreviated version of the CES-D 20-item instrument, which consists of self-reported items used to identify populations at high risk of having major depression. The CES-D8 is commonly used in epidemiological studies of older adults^15^-^20^ and has excellent reliability and validity across a range of older populations (average Cronbach alpha = 0.84).^18^ Respondents were asked whether, during the past week, most of the time they felt depressed, thought everything was an effort, found sleep restful, were happy, felt alone, enjoyed life, felt sad, or felt they could not move forward.^14^,^20^ The answer options were yes or no, with a maximum score of 8.

#### Self-rated health

SRH was assessed through the following question: “In general, how would you evaluate your health?” Participants could answer (1) Very good or excellent, (2) Good, (3) Regular, (4) Bad, or (5) Very bad. This variable was dichotomized into two categories: I) Very good or excellent, good, or average (encompassing alternatives 1 through 3); and II) Poor or very poor (encompassing alternatives 4 and 5).

#### Number of chronic illnesses

We assessed chronic illnesses by adding the number of positive responses to the question “Has any doctor ever told you that you have…” for 15 conditions commonly found in older populations, namely: hypertension, diabetes, dyslipidemia, heart attack/angina, heart failure, stroke, asthma, chronic obstructive pulmonary disease (COPD), arthritis, osteoporosis, chronic back problems, cancer, chronic kidney failure, Parkinson disease, and Alzheimer’s disease.

#### Other measures

Sociodemographic variables (age, sex, education – illiterate or minimal education – up to year 4), whether the respondent currently has a partner, and information about unhealthy behaviors (tobacco smoking status and current alcohol consumption) were recorded.

### Statistical analysis

Multivariate analysis using logistic regression was used to estimate the association of depressive symptoms with poor or very poor SRH. First, the multivariate analysis was adjusted by the sociodemographic characteristics (age, sex, having a partner, educational level); then, unhealthy behavior variables (current alcohol consumption and tobacco smoking) were added into the second model, before the number of chronic illnesses was added to produce the final model. We tested our data for the presence of multicollinearity and found a mean variance inflation factor (VIF) of 1.16, demonstrating our estimates were not inflated. An odds ratio (OR) with 95% CIs was calculated for each of the variables used in the models. Statistical significance was set at p < 0.05. Statistical analysis was conducted using Stata^®^ version 14.

## Results

Of the initial sample of 9,412 participants, 1,558 were excluded due to missing data (1,109 for depression, 20 for SRH, and 459 for the other covariates). Table 1 shows the data from the 7,924 included respondents. Of these, 928 rated their health as poor or very poor, representing 11% of the sample.

The poor or very poor SRH group comprised mostly women (57.9%), with a mean age of 63.6 years. Most had a partner (62.3%) and were illiterate or had minimal formal education (68.3%). Regarding unhealthy behaviors, 15.7% reported currently using alcohol and 21.7% were active smokers. Those who rated their health as poor or very poor had a significantly higher number of depressive symptoms compared with those who rated their health as very good or excellent, good, or average (4.8 vs. 2.6).

Table 2 shows the results of the univariate and multivariate analyses. We found that female sex was not associated with poor or very poor SRH compared to male sex (OR 1.08, 95%CI 0.94-1.24). After adjusting for sociodemographic factors, unhealthy behaviors, and number of chronic conditions, however, women were less likely to report poor or very poor SRH (OR 0.50, 95%CI 0.42-0.59). The association between age and poor or very poor SRH was not significant even after adjusting for other factors in the models (OR 0.99, 95%CI 0.98- 0.99). Being illiterate or having little formal schooling were significantly associated with poor or very poor SRH (OR 2.26, 95%CI 1.95-2.62), and this association remained even after controlling for the other variables in the third model (OR 1.88, 95%CI 1.59-1.22). Having a partner did not appear to be associated with poor or very poor SRH (OR 0.98, 95%CI 0.84-1.16) (Table 2).

Individuals who reported alcohol consumption at the time of evaluation had lower odds of considering their health as negative, even after the third adjustment model, compared to those who did not consume alcohol (OR 0.46, 95%CI 0.37-0.57). Compared to non-smokers, being an active smoker or being a former smoker was significantly associated with poor or very poor SRH in the three adjusted models (OR 1.42, 95%CI 1.15-1.77) (Table 2).

Having depressive symptoms and a greater number of chronic illnesses had a significant magnitude of association with poor or very poor SRH. The association remained strong even after adjusting for all the other variables. The magnitude of the association between the presence of depressive symptoms and poor or very poor SRH was reduced when the number of chronic illnesses was included in the multivariate analysis, along with the other sociodemographic variables and unhealthy behaviors (OR 1.35, 95%CI 1.31-1.39) (Table 2).

Individuals with a greater number of chronic illnesses had higher odds of reporting poor or very poor SRH than those who had no reported illnesses (OR 1.51, 95%CI 1.46-1.57) in the unadjusted analysis. In the third adjusted model, there was a small reduction in the magnitude of such an association (OR 1.43, 95%CI 1.37-1.49) (Table 2).

## Discussion

We found an overall prevalence of poor SRH of 11%, which is below the average found in a systematic review of Brazilian literature on SRH and associated factors in older adults (from 12.6 to 51.9%). However, there were variations across these studies regarding how SRH was dichotomized as good or poor. The average or fair category was sometimes grouped with positive responses (good, very good), and sometimes with negative responses (poor, very poor).^21^ The strong and independent association between depressive symptoms and SRH is well established in cross-sectional studies and confirmed by longitudinal studies. Jang et al.^22^ evaluated older Korean Americans for 2 years and demonstrated that, regardless of physical health status and its changes, elevated depressive symptoms played a substantial role in predicting the decline in subjective health perception.

The interpretation that depression can worsen SRH status can be mediated by Beck’s cognitive model of depression,^23^ whereby individuals with depression are likely to selectively perceive and report negative stimuli (biased attention) and have negative internal representations about the self and the environment. Depressed older adults often focus their complaints on physical symptoms, minimizing the emotional aspects of any mental disorder.^7^ Negative perceptions about illnesses, such as viewing the illness as chronic, cyclical, or uncontrollable, or as having serious consequences, are significantly associated with depression across several medical conditions.^24^ In addition, growing evidence suggests that everyday interpretations of symptoms, such as catastrophizing (believing in worst-case scenarios) about symptoms or viewing symptoms as signs of biological damage, are also key correlates of depression in physical illness.^25^ It is important to note that depression in older people often occurs in the context of clinical comorbidities. In our third model of multivariate analysis, having chronic diseases reduced the magnitude of the association between poorer SRH and depression, showing the influence of chronic diseases on the perception of health of this population. Depressed older adults are large consumers of medical care and account for a disproportionately high number of primary care visits compared to those without depressive symptoms. It has been shown that having both poor SRH and depressive symptoms are significantly associated with greater utilization of outpatient services. One study reported that for each point increase in CES-D score, the number of outpatient visits increased by 3%.^26^ Future studies should therefore focus on analyzing treatment history of depression, medication use, and hospitalizations of patients diagnosed with depression in relation to their SHR.

Research shows that poor SRH is more frequent among women, who have a higher prevalence of chronic conditions with a low mortality rate, such as arthritis and depression. In addition, women use health services more often than men, which can indicate a higher likelihood of receiving a diagnosis of existing chronic conditions compared to men.^27^ Women live longer, which makes them more likely to experience more years of functional decline than men^28^ and, with advancing age, there is a tendency for health status to deteriorate; thus, worsening SRH is to be expected. For example, in a Brazilian study using data from the 2003 Pesquisa Nacional por Amostra de Domicílios (PNAD) that assessed determinants of SRH status, increasing age was associated with a higher propensity to reporting poor or very poor SRH.^29^ Although some studies have found aging to be associated with more positive SRH, this may be due to a reduction in expectation regarding health status among the oldest old, as simply surviving up to that stage can be already perceived as having a positive state of health compared to those who died younger. It is also possible that more positive SRH in later life is related to the effect of selective survival, whereby only the healthiest individuals are likely to live longer, and, therefore, experience better SRH.^30^,^31^ Future studies comparing demographic and behavioral characteristics with the presence of depressive symptoms among men and women at the same age would be helpful to assess the impact of these variables on different sex groups.

The association between low educational attainment, illiteracy, and SRH is well established in the literature; the percentage of health self-assessed as poor was higher among individuals with fewer years of schooling.^32^,^33^ Introducing variables related to health behaviors and chronic conditions in the second and third adjusted models reduced, but did not eliminate, the effect of the association between educational attainment and poor or very poor SRH, suggesting that individuals with higher educational attainment are more likely to adopt healthy behaviors and potentially more able to prevent chronic conditions.^34^

It has been suggested that the reduction in alcohol intake commonly seen in older groups compared to younger groups is associated with reduced risk for coronary heart disease, which result in more positive SRH.^35^ Another possible explanation for the lower odds of alcohol consumers considering SRH as negative at the time of the evaluation is that individuals who have good SRH are less concerned about reducing or stopping their alcohol consumption; however, further studies are needed to investigate this association.^31^ Frisher et al.^35^ assessed the association between alcohol intake and poor SRH among people aged 50 and above and found that poorer SRH was more prevalent among those who did not consume alcohol (ceased use or never used it). Despite these findings, alcohol-related mortality is also highest in older age groups and intake is increasing among older adults. Former and current smokers were more likely to have poorer SRH than those who had never smoked, which is consistent with other cross-sectional studies^36^ and is in line with a longitudinal study of adults aged 50 and over, in which smoking was associated with lower SRH.^37^

Analysis of these determinants of SRH allowed us to formulate some hypotheses which, in the future, can be better confirmed in longitudinal studies. The strength of this study is the use of a large representative sample of non-institutionalized Brazilian older adults; it is one of the few studies that has assessed the relationship between depressive symptoms and SRH. Some limitations should be noted, such as the cross-sectional design, which precluded establishment of causal relationships between the variables of interest.^14^ Another limitation is the use of the short-form CES-D8 scale, which is a screening tool that allows detection of clinically significant depressive symptoms but maybe be limited as a measure of major depression. However, evidence is accumulating that depressive symptoms in older persons, even in the absence of major depression (subsyndromal depression), may have a significant impact on health and functioning.^18^,^37^ Chronic diseases were evaluated by self-reporting against a list of diseases, taking into account only individuals who had access to the health system and had received a diagnosis for such diseases.^38^ There are several variables directly related to one’s perceptions of health, such as functional ability and the frailty syndrome,^30^,^37^ which share common symptoms and can overlap with depression. However, we decided to use the number of health conditions as a general proxy measure to account for overall losses in intrinsic capacity, which are commonly present in these other conditions.

In conclusion, SRH is a multidimensional construct related to chronic conditions, as well as demographic and socioeconomic variables and unhealthy behaviors. Having depressive symptoms is strongly associated with having a more negative SRH, regardless of the presence of chronic conditions and other factors, showing the impact of this construct on general health. Considering that depressive symptoms in older adults constitute a potentially modifiable and preventable condition,^39^,^40^ our findings support the implementation of early intervention strategies to promote a positive perception of one’s own health.

## Disclosure

The authors report no conflicts of interest.

## Figures and Tables

**Table 1:** Sample characteristics according to self-rated health (n=7,924)

		Self-rated health		
	Total (n=7,924)	Bad or very bad (n=928)	Very good or excellent, good or average (n=6,996)	p-value (χ^2^ or *t* test)
Depressive symptoms	2.8 (2.5)	4.8 (2.6)	2.6 (2.3)	< 0.001
Sex (female)	4,449 (53.9)	536 (57.9)	3,913 (53.4)	0.067
Age (mean, SD)	62.6 (9.4)	63.6 (9.6)	62.5 (9.4)	0.099
Having a partner	4,716 (65.1)	528 (62.3)	4,188 (65.5)	0.117
Illiterate or minimal education	4,176 (48.8)	647 (68.3)	3,529 (46.4)	< 0.001
Current alcohol consumption	2,224 (30.8)	136 (15.7)	2,088 (32.6)	< 0.001
Tobacco smoking				< 0.001
Never smoked	3,581 (45.3)	349 (36.8)	3,232 (46.4)	
Former smoker	2,977 (37.3)	384 (41.5)	2,593 (36.8)	
Current smoker	1,366 (17.3)	195 (21.7)	1,171 (16.8)	
Number of chronic conditions	2.2 (1.7)	3.6 (1.6)	2.0 (1.6)	< 0.001

Data presented as n (%), unless otherwise specified.

**Table 2:** Association of depression and other sociodemographic and health related variables with poor or very poor self-rated health (n=7,924)

	Crude	Adjusted†	Adjusted‡	Adjusted§
Depressive symptoms	1.40 (1.36-1.44)	1.42 (1.38-1.46)	1.41 (1.37-1.46)	1.35 (1.31-1.39)
Age (years)	1.00 (1.00-1.01)	1.01 (1.00-1.01)	0.99 (0.98-1.01)	0.99 (0.98-0.99)
Sex (female)	1.08 (0.94-1.24)	0.70 (0.60-0.82)	0.65 (0.56-0.77)	0.50 (0.42-0.59)
Having a partner	0.89 (0.77-1.02)	1.08 (0.93-1.26)	1.07 (0.91- 1.24)	0.98 (0.84-1.16)
Illiterate or minimal education	2.26 (1.95-2.62)	2.03 (1.73-2.38)	1.83 (1.56-2.15)	1.88 (1.59-1.22)
Current alcohol consumption	0.40 (0.33-0.49)	-	0.45 (0.36-0.55)	0.46 (0.37-0.57)
Tobacco smoking		-		
Never smoked	1.00		1.00	1.00
Former smoker	1.37 (1.17-1.60)		1.31 (1.11-1.55)	1.26 (1.06-1.49)
Current smoker	1.54 (1.28-1.86)		1.35 (1.10-1.66)	1.42 (1.15-1.77)
Number of chronic conditions	1.51 (1.46-1.57)	-		1.43 (1.37-1.49)

† Adjusted for age, sex, having a partner, low educational level.

‡ Adjusted for age, sex, having a partner, low educational level, current alcohol consumption, sedentary lifestyle, tobacco smoking.

§ Adjusted for age, sex, with having partner, low educational level, current alcohol consumption, sedentary lifestyle, tobacco smoking, number of chronic conditions.
